# Animated Randomness, Avatars, Movement, and Personalization in Risk Graphics

**DOI:** 10.2196/jmir.2895

**Published:** 2014-03-18

**Authors:** Holly O Witteman, Andrea Fuhrel-Forbis, Harindra C Wijeysundera, Nicole Exe, Mark Dickson, Lisa Holtzman, Valerie C Kahn, Brian J Zikmund-Fisher

**Affiliations:** ^1^Department of Family and Emergency MedicineFaculty of MedicineLaval UniversityQuebec City, QCCanada; ^2^Office of Education and Continuing Professional DevelopmentFaculty of MedicineLaval UniversityQuebec City, QCCanada; ^3^Research Centre of the CHU de QuébecQuebec City, QCCanada; ^4^Center for Bioethics and Social Sciences in MedicineUniversity of MichiganAnn Arbor, MIUnited States; ^5^Research Center for Group DynamicsInstitute for Social ResearchUniversity of MichiganAnn Arbor, MIUnited States; ^6^Schulich Heart CentreSunnybrook Health Sciences CentreToronto, ONCanada; ^7^Division of CardiologyDepartment of MedicineUniversity of TorontoToronto, ONCanada; ^8^Toronto Health Economics and Technology Assessment (THETA) CollaborativeUniversity of TorontoToronto, ONCanada; ^9^ReThink HealthFannie E Rippel FoundationMorristown, NJUnited States; ^10^Department of Health Behavior and Health EducationSchool of Public HealthUniversity of MichiganAnn Arbor, MIUnited States; ^11^Department of Internal MedicineUniversity of MichiganAnn Arbor, MIUnited States; ^12^Risk Science CenterSchool of Public HealthUniversity of MichiganAnn Arbor, MIUnited States

**Keywords:** risk graphics, health communication, cardiovascular disease, animation, avatar, pictograph, icon array

## Abstract

**Background:**

Risk communication involves conveying two inherently difficult concepts about the nature of risk: the underlying random distribution of outcomes and how a population-based proportion applies to an individual.

**Objective:**

The objective of this study was to test whether 4 design factors in icon arrays—animated random dispersal of risk events, avatars to represent an individual, personalization (operationalized as choosing the avatar’s color), and a moving avatar—might help convey randomness and how a given risk applies to an individual, thereby better aligning risk perceptions with risk estimates.

**Methods:**

A diverse sample of 3630 adults with no previous heart disease or stroke completed an online nested factorial experiment in which they entered personal health data into a risk calculator that estimated 10-year risk of cardiovascular disease based on a robust and validated model. We randomly assigned them to view their results in 1 of 10 risk graphics that used different combinations of the 4 design factors. We measured participants’ risk perceptions as our primary outcome, as well as behavioral intentions and recall of the risk estimate. We also assessed subjective numeracy, whether or not participants knew anyone who had died of cardiovascular causes, and whether or not they knew their blood pressure and cholesterol as potential moderators.

**Results:**

Animated randomness was associated with better alignment between risk estimates and risk perceptions (*F*_1,3576_=6.12, *P*=.01); however, it also led to lower scores on healthy lifestyle intentions (*F*_1,3572_=11.1, *P*<.001). Using an avatar increased risk perceptions overall (*F*_1,3576_=4.61, *P*=.03) and most significantly increased risk perceptions among those who did not know a particular person who had experienced the grave outcomes of cardiovascular disease (*F*_1,3576_=5.88, *P*=.02). Using an avatar also better aligned actual risk estimates with intentions to see a doctor (*F*_1,3556_=6.38, *P*=.01). No design factors had main effects on recall, but animated randomness was associated with better recall for those at lower risk and worse recall for those at higher risk (*F*_1,3544_=7.06, *P*=.01).

**Conclusions:**

Animated randomness may help people better understand the random nature of risk. However, in the context of cardiovascular risk, such understanding may result in lower healthy lifestyle intentions. Therefore, whether or not to display randomness may depend on whether one’s goal is to persuade or to inform. Avatars show promise for helping people grasp how population-based statistics map to an individual case.

## Introduction

Health risk communication is an inherently challenging proposition. People’s risk perceptions are shaped by powerful cognitive and affective biases [1,2] and often align poorly with their actual risk [3-5], even when they are provided with accurate risk estimates [6]. In other words, people at low risk may feel at considerable risk, whereas people at high risk may not perceive themselves as such.

Lack of alignment between actual and perceived risk may be partly due to barriers to comprehension, such as low health literacy or, in the case of communication about numbers, low numeracy [7,8]. Across levels of education and expertise, many people, particularly those with poor numeracy, have trouble interpreting numbers in health risk communications [9,10] and demonstrate biased interpretations of proportions [11,12].

Icon arrays (or pictographs) are graphical displays, often with 100 or 1000 icons arranged in rows and columns and in which each icon represents one unit in the population of interest. They have been shown to help people overcome natural tendencies toward misinterpretation [13] and, for people with low numeracy, maximize comprehension compared to other graphic types and text or numbers alone [14-18]. However, despite their advantages, they can still fall short of facilitating full comprehension [19].

One of the key challenges to such comprehension is adequately conveying the inherent uncertainty of risk statistics. In this study, we aimed to address the issue of aleatory or first-order uncertainty, which has been highlighted as an entrenched conceptual problem and a key challenge when communicating risk. First-order uncertainty arises from the “fundamental indeterminacy” of future events [20]. We operationalized the communication of such indeterminacy as consisting of 2 related challenges: conveying the randomness of events and helping people grasp how population-based statistics map onto individual circumstances.

Randomness is conceptually challenging, especially for people with little training in statistics. For example, many people believe that their iPod’s shuffle feature does not actually choose songs randomly because the algorithm may play several songs from the same album in a row, or the user may not hear a new song within their expected time frame. These perceptions persist even though such behaviors on the part of the algorithm are perfectly reasonable within a random ordering [21]. In health communication, previous work suggests that using randomly dispersed events in pictographs may help to better convey the randomness inherent in population-based risk estimates; however, this work also suggests that people may find such graphics confusing, may find the estimate less certain, and may have a more difficult time interpreting the magnitude of the risk when it is scattered randomly in the display [22-28]. Our research group previously tested methods of simultaneous animated randomness in the context of 2 side-by-side icon arrays and found, similarly, that many methods of displaying randomness resulted in confusion, but that at least one method had promise [29].

In addition to the challenge of interpreting the meaning of background randomness, it is difficult for people to map population-based statistics, which are often proportions, onto individual circumstances, which are often whole numbers. No matter how average they might be, a family cannot, after all, actually have 2.3 children [30]. When it comes to health risks, it can be conceptually difficult to apply the information that a side effect occurs 16% of the time and is randomly distributed within a population to an individual’s binary experience of either having the side effect or not. Although less work in health risk communication has focused on this issue than on randomness, a gamified design—in which participants clicked concealed icons in an array to reveal whether or not the event occurred for each individual in the population—showed promise, with a positive trend toward helping people understand the risks [31].

In this study, we considered another potential approach for helping people understand what population-based statistics mean for individual risk: the use of avatars. People understand avatars to represent individuals and react to them accordingly; for example, by putting more trust in more relatable avatars [32] or those with a more professional appearance [33]. Further, people perceive their avatar as representing them [34-36] and have been shown to identify strongly with their avatars in a variety of online applications [37-39]. The phenomenon of integrating one’s avatar into one’s identity, dubbed the Proteus Effect, is demonstrated in the way that social gaming players exhibit gender role behavior that aligns more strongly with the gender of their avatar than with their actual gender identity [40] and in experimental studies where people who are assigned a taller or more attractive avatar in a simulation subsequently show more confident or more intimate behavior in a face-to-face interaction [41].

In this study, we evaluated 4 specific risk graphic design factors dealing with the display of randomness and the use of avatars that, in principle, might better convey these challenging concepts—the randomness of events and how population-based statistics apply to an individual—with the goal of helping people better understand the nature of a health risk.

In addition to these experimental factors, we also examined the potential moderating effects of 3 planned individual factors: (1) an individual’s actual level of risk, (2) his or her numeracy, and (3) whether or not she or he has known someone who has experienced the negative health event in question. Each of these may influence how people respond to different risk graphics by moderating the personal salience of the risk information or the way people understand risk numbers.

Regarding the first moderator, to our knowledge, no literature exists concerning the different responses to the same risk graphic formats at different levels of risk. However, it seems plausible that someone receiving a low risk estimate might respond to a particular risk presentation format differently than someone receiving a high risk estimate. Regarding the second moderator, different risk graphic formats have been shown to generate different responses in people at different levels of numeracy [15]. Most importantly, a previous study by our group that set the stage for this study demonstrated, among other findings, that interactive elements were associated with lower understanding among people with higher numeracy, but not among those with lower numeracy [29]. Regarding the third moderator, we speculated that personal familiarity with the negative health outcomes being presented (eg, knowing someone who has experienced grave outcomes) might provide a concrete personal example of how a statistical probability can map onto individual circumstances and, thus, make the risk more salient. People are more sensitive to risks associated with events that they can more easily call to mind [42] or that have stronger emotions associated with them [43] (eg, due to personal loss).

We further investigated the effects of 2 additional potential moderators whose importance emerged from our observed data: whether or not people know their (1) blood pressure and (2) cholesterol and are thus able to choose from a drop-down menu of potential ranges for such values. Although this information was not required in order for participants to receive a risk estimate, having entered more information relevant to one’s own individual health may well increase the salience of the risk information.

Ultimately, our aim is to improve understanding of risk estimates, which we operationalized in this study as alignment between subjective risk perception and an objective risk estimate along with accurate recall of the risk estimate. Therefore, we investigated the following specific research questions: (1) which design factors might help to increase alignment between perceptions of risk and actual risk, (2) which design factors might help to encourage intentions toward actions associated with healthy living, and (3) do any of the design factors affect recall of risk numbers? The first and third of these specifically addressed our primary goal of improving comprehension; the second addressed questions around the applicability of these design factors to different purposes.

## Methods

### Recruitment

We invited a random sample of US adults aged 35 to 74 years from a panel of Internet users administered by Survey Sampling International (SSI), stratified by gender, age, and race to ensure demographic diversity in similar proportions to the US population, to participate in an online survey. The number of email invitations sent to each stratum was dynamically adjusted to maintain demographic balance despite varying response rates. Participants who completed this experiment as well as another unrelated cross-randomized study contained within the same survey were entered into both an instant-win contest and a monthly drawing administered by SSI for modest prizes. The study was deemed exempt by the University of Michigan Health Sciences and Behavioral Sciences Institutional Review Board as anonymous survey research. All participants viewed a consent page before clicking to begin the study in which they were informed that the survey would involve learning about their personal risk of heart disease and stroke, and that if they did not want to learn about their risk, they should not participate in the study. At the conclusion of the survey, participants were provided with a list of resources for learning more about cardiovascular health and ways to prevent cardiovascular disease.

### Design of Experiment

To explore our research questions, we chose the clinical context of general cardiovascular disease because of the availability of a robust simple model [44] that estimates an individual’s risk of general cardiovascular disease within the next 10 years based largely on information that laypeople would be likely to know. Namely, it allows for the input of height and weight if blood lipids results are not known. It is widely applicable and returns a large range of risk numbers, thus providing a fruitful context for investigating risk communication methods. The model was originally developed for clinical use and uses blood pressure as one of the predictors. In our study, because we were deploying the model in a survey of laypeople, we allowed for the fact that people may not know their blood pressure. When people indicated this was the case, we made conservative assumptions. If these participants responded that they were not taking any medication to treat high blood pressure, we assigned the lowest possible number of model points (ie, lowest risk) using average blood pressure for the person’s age. If they responded that they were taking medication to treat high blood pressure, we allotted 2 model points from the potential ranges of 0 to 5 for men and -1 to 7 for women (higher points mean higher risk). This corresponds to a treated systolic blood pressure between 120 and 129 mm Hg.

Estimates returned by the model range from a risk of less than 1% to a risk greater than 30%. Risk estimates between the lower and upper limits are returned as integer percent values. In other words, the vast majority of risk estimates were numbers such as 4% or 21%, but results at the upper and lower ends of the range were not simple integers. For the risk graphics, we described these less specific values in the legend and introductory text as being “less than 1%” and “more than 30%,” and used 1 and 31 event rectangles, respectively, in the array of 100. Similarly, in our analyses, we used the values .999 and 31 as conservative estimates of these cases, respectively. Throughout the experiment, rather than the term, “general cardiovascular disease,” we used the more familiar terminology “heart disease and stroke.”

The model applies only to people who have not already experienced general cardiovascular disease, so we screened out those who had a history of cardiovascular disease (448/4124, 11%). Remaining participants were asked to enter their information to calculate their personal risk estimate, which they subsequently viewed in an animated risk graphic randomly assigned from 10 possible versions created from the 4 risk graphic design factors (see Design of Risk Graphics section). We only presented absolute numbers and did not provide any context of expected levels of risk. In other words, we did not give participants any indication of whether their risk was higher or lower than might be expected for their age and gender before we assessed their risk perceptions.

### Design of Risk Graphics

For all designs, we used a 10×10 matrix of rectangle icons and animated the construction of the icon array (as previously tested [29]) to visually introduce each event rectangle one at a time. This animation served to signal several concepts to people viewing the risk graphic. First, it highlighted the discrete and, hence, countable nature of events within the population of 100. This is a strength of both natural frequency and icon array formats. Second, it served as a temporal signal about the size of an individual’s risk. A person given the risk estimate “1 in 100” had only to wait briefly for the 1 event rectangle representing a risk event to appear before moving on. By contrast, someone whose risk was “30 in 100” had to wait 30 times longer for the animation to add all the event rectangles. Signaling is frequently used in multimedia to draw attention to important ideas and elements [45,46].

In keeping with our goal of conveying 2 key concepts (ie, the underlying randomness of events and the mapping of population-based statistics onto individuals), we used 2 main experimental design factors in our graphics. The first of these was animated randomness, a factor that showed promise when previously explored in combination with a different set of design factors [29]. Participants randomized to graphics with the random design factor observed the event rectangles appear one at a time as in the standard condition, but in a random spatial position throughout the array. Once all event rectangles appeared, the animation concluded with all the randomly dispersed event rectangles settling into a standard grouped display. The goal of this settling was to avoid the comprehension problems observed in previous research on randomly dispersed events in icon arrays [22-29].

The second main design factor was the use of an avatar. We designed this factor to give more explicit signals about how such risks apply to a single individual. Graphics that included a standard avatar had a generic avatar shape animated to drop into the icon array and disappear, then emerge with a question mark at the conclusion of the animation. The disappearance and re-emergence with a question mark were intended to convey that we do not know which event out of the 100 will apply to a single individual. Within the avatar design factor, we also had 2 other nested factors. The first of these (avatar moves) specified that after the avatar was dropped into the array, it would move within the array, randomly landing on either event rectangles or nonevent rectangles to further emphasize how the randomness inherent in the risk statistic applies to a single individual. The second nested factor (color choice), designed to help participants identify with the avatar, offered participants the chance to choose a different color from a palette of Web colors, instead of the default color, which was standard Web black (#000000).

**Figure 1:**
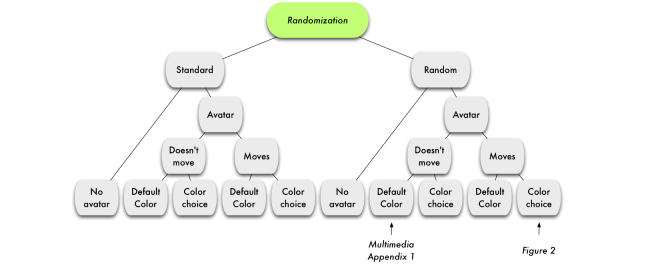
Randomization and graphics factors.

**Figure 2:**
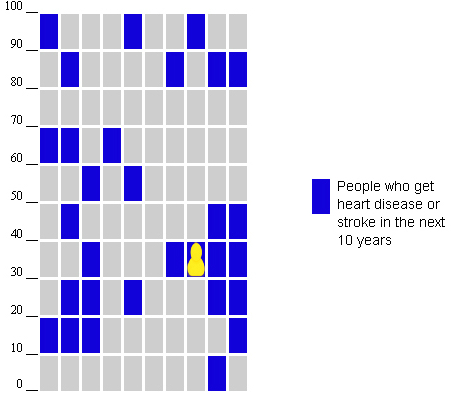
Sample risk graphic (random, avatar moves, color choice).

These factors created a 2×2 factorial design nested within another 2×2 factorial design. See Figure 1 for a chart illustrating the experimental design, and Figure 2 for a still image of a sample graphic. See Multimedia Appendix 1 to view a video (.mp4 version) of a graphic with the random factor and a nonmoving default color avatar (see Multimedia Appendix 2 for the same video in .avi format).

### Technologies

We programmed the risk graphics and an avatar color-chooser in ActionScript 3.0 and integrated them into a custom survey system programmed in Ruby on Rails. The number of event rectangles in the icon array were set dynamically via JavaScript, using participants’ risk numbers as calculated by the model algorithm implemented in the survey system.

### Measures

#### Independent Variables

There were 4 independent dichotomous variables. The random variable describes whether or not the event rectangles were dispersed randomly in the pictograph (random condition) or whether they are grouped together at the bottom of the page (standard condition). The avatar variable describes whether or not an avatar was used in the risk graphic. The avatar moves variable indicates whether or not the avatar moved around randomly within the pictograph, randomly landing on event rectangles or not, as the animation proceeded. The color choice variable refers to whether or not participants were asked to select a color for the avatar that they felt best represented themselves.

#### Primary Outcome

The primary outcome variable for this study, risk perception, was created from 3 questions, all asked together on the same page immediately after viewing the risk graphic. These questions were intended to capture people’s immediate reactions to the risk number and graphic presentation. We first asked participants to answer the question, “How big or small does this risk feel to you?” on a 10-point Likert scale with anchors “extremely small” on the left and “extremely big” on the right. We then asked people to indicate, “How worried do you feel about your chance of getting heart disease or stroke in the next 10 years?” on a 10-point Likert scale anchored by “not at all worried” on the left and “extremely worried” on the right. Values for 10-point Likert scales were assigned as 0-9 but survey responses were not labeled numerically, meaning that participants did not see any numbers, only a horizontal visual array of equally-spaced radio buttons. Finally, we asked them, “How likely does it feel to you that you will actually get heart disease or stroke in the next 10 years?” which we assessed on a horizontal slider. The slider recorded integer values between 0 (label “extremely unlikely”) and 100 (label “extremely likely”). Participants saw only the visual position of the slider, not the numeric values representing their response. Because we wanted to capture participants’ subjective risk sense, we used this measure rather than asking for a numeric estimate of their risk. We surmised that if we asked for a numeric estimate, many participants would simply return the risk estimate they had been given. We further suspected that this would be most likely to occur among participants with higher numeracy; thus, this measure could bias the potential effects of numeracy on the subjective feeling of being at risk. To combine these 3 measures with equal weight accorded to each, we rescaled the likelihood question by multiplying values by 9/100. We then averaged responses to the 3 questions (Cronbach alpha=.88) to calculate risk perception.

#### Secondary Outcomes

We considered 3 secondary outcome variables in this study: 2 behavioral intention measures and a recall task.

Behavioral intentions were all collected together on 1 page. Participants were given the text, “There are ways to improve your heart health and reduce your risk of heart disease and stroke. How likely are you to do the following things in the next 30 days?” This was followed by a list of 4 or 5 potential actions: quit smoking (presented only to participants who indicated in the risk calculator they had smoked in the last month); exercise 30 to 60 minutes a day, at least 5 days a week; eat a diet that is low in salt, low in fat, and has at least 5 to 10 servings of fruits and vegetables each day; start a weight loss program; and make an appointment to see a doctor about your heart health. Responses were collected for each action on a 10-point Likert scale with anchors “not at all likely” on the left and “extremely likely” on the right. Again, responses were not labeled with their numeric value, meaning that participants did not see any numbers, only a horizontal visual array of equally-spaced radio buttons. Participants were not provided with details about these behaviors beyond their verbal label, nor were they given any information about the extent to which engaging in such behaviors might lower their risk. At the conclusion of the survey, after the study was complete, participants were provided with links to webpages by reputable sources about healthy lifestyles for reducing cardiovascular risk. (See also Multimedia Appendix 3 for results of a small secondary study that was conducted within this study about the effects of a “heart age” message on behavioral intentions.)

The first 4 behavioral statements (3 for nonsmokers) are typical behavioral outcomes in interventions addressing cardiovascular health. We averaged them to form the lifestyle intentions scale (nonsmokers: Cronbach alpha=.68; smokers: Cronbach alpha=.70). We added the final variable, see a doctor, because we postulated that intentions to see a doctor for personalized counsel would be a more appropriate measure of the effects of a brief online risk calculator. In other words, an increased understanding of one’s risk may not be sufficient to provoke behavior change, but it may prompt people to seek more information via a medical consultation.

Recall was collected on the last page of the survey (participants had been presented with 14 to 22 pages since receiving their risk estimate) by asking participants, “Please answer the following question based on your memory of the numbers you were given by the risk calculator earlier in this study. If you are unsure, please take your best guess. Please do not go back to check your answers. If there were 100 people exactly like you, how many of them would have heart disease or stroke in the next 10 years?” Participants entered their recalled value in a text box. To analyze the effects of experimental and moderating factors on recall, we defined the dependent variable recall as the absolute difference between the recalled estimate and the correct value. However, to maximize clarity for readers when tabulating descriptive results about recall in this paper, we define correct recall as a recalled risk within 5 percentage points in either direction of the risk estimate.

#### Moderators

We planned for the inclusion of 3 attributes in our model that might moderate participants’ responses. First, we considered the impact of the participant’s actual estimated 10-year risk of cardiovascular disease as presented to them (actual risk). We used the original quasi-continuous variable in our analyses, but to facilitate readers’ interpretation of descriptive statistics in this paper, we present data according to whether a participant’s risk was below the median risk (8%) or not. We further distinguish participants at either end of the spectrum of risk estimates for whom the model provided a less precise numeric risk estimate. Thus, the levels for reporting are very low risk (<1%), lower risk (1%-7%), higher risk (8-30%), and very high risk (>30%). We emphasize that these labels were not shown to study participants nor were they used for analysis; they are simply for readers’ comprehension. We further note that because so few participants were in the very low risk group (n=7; see Table 1), in the Results sections of this paper, we report mean values only for the latter 3 risk levels: lower risk, higher risk, and very high risk.

We collected 2 self-report individual difference measures, selected because of their potential moderating effect on individuals’ responses to different ways of presenting risk numbers and graphics about cardiovascular disease. Participants completed a validated measure of numeracy, the Subjective Numeracy Scale, which asks people how confident they feel with numbers and how much they prefer information be presented numerically [47,48]. We also asked participants an ad hoc question, “Have you ever known anyone who died of heart problems or stroke?” to assess their personal familiarity with the potential impact of cardiovascular disease. We hypothesized that familiarity would provide a concrete personal example of how a statistical probability can map onto individual circumstances.

In addition to these planned moderators, we noted in our data that a sizeable proportion of participants indicated that they did not know their blood pressure and/or cholesterol. Given that the input of such personal information might affect the salience of the risk, we also included 2 additional variables, blood pressure known and cholesterol known, in our analyses. For the latter, we classified participants who knew either 1 or both of their total or high-density lipoprotein (HDL) cholesterol as knowing their cholesterol.

### Statistical Analyses

The effects of risk graphic factors and individual difference measures on outcomes were examined via nested factorial ANOVA. All main effects were analyzed, as were all possible interactions. For the primary outcome, we used an alpha level of .05. For the 3 secondary outcomes, to control for Type I error, we applied a Bonferroni correction, yielding an alpha level of .017. All tests were 2-tailed. To present results, we give *F* statistics and *P* values, as well as mean values to provide a sense of the size of differences observed. Exploratory correlations were calculated as Pearson’s correlations. Data were entered and analyzed in R, version 2.15.2 [49] with use of the package psy, version 1.1 [50], to calculate Cronbach alpha scores, and the package car, version 2.0-18 [51] for conducting Levene’s test of homogeneity of variances. Because participants whose actual risk was less than 1% or greater than 30% were not given an exact number in the text (although they were given an exact number of event rectangles in the graphic), we conducted analyses on all outcomes both with and without those participants to explore the impact of the upper and lower limits of the underlying risk model on our findings.

## Results

### Study Participants

Of the 4859 people who received an invitation email and clicked the link to the survey, 4124 (85%) completed the survey. Of these, 3676 (89%) were eligible for this study, meaning that they were between ages 35 and 74 years and had neither been diagnosed with heart disease nor had a stroke. For analysis, we included participants who completed the full survey, which included this study, a second unrelated study, demographic questions, and other measures of individual differences. The median time to complete the full survey was 16 minutes and the interquartile range (IQR) was 11 minutes. We excluded participants who completed the full survey in less than 6 minutes from analysis because this speed suggested that they may not have been paying attention to the content. Thus, the final sample for analysis comprised responses from 3630 people (99% of eligible respondents).

Participants were diverse in terms of gender, age, ethnicity, race, and level of education. The median 10-year risk of general cardiovascular disease was 8% (IQR 11%). See Table 1 for details of study participant characteristics. None of these characteristics varied significantly between the different graphics (all *P*>.05).

**Table 1:** Study participant characteristics (N=3630).

Characteristic			Statistic
Age (years), mean (SD)			53 (10)
**Gender, n (%)**			
	Female		2000 (55)
	Male		1630 (45)
**Ethnicity, n (%)**			
	Hispanic		404 (11)
	Middle Eastern		44 (1)
**Race, n (%)**			
	White or Caucasian		2827 (78)
	Black or African American		514 (14)
	American Indian or Alaska Native		48 (1)
	Asian or Asian-American		145 (4)
	Pacific Islander or Native Hawaiian		10 (<1)
	Other		124 (3)
**Highest education level reached, n (%)**			
	None		1 (<1)
	Elementary school		3 (<1)
	Some high school, but no diploma		73 (2)
	High school (diploma or GED)		681 (19)
	Trade school		216 (6)
	Some college, but no degree		975 (27)
	Associate’s degree (eg, AA, AS)		384 (11)
	Bachelor’s degree (eg, BS, BA)		871 (24)
	Master’s degree (eg, MA, MPH)		335 (9)
	Doctoral/professional degree (eg, PhD, MD)		88 (2)
General cardiovascular disease 10-year risk, median (IQR)			8 (11)
**General cardiovascular disease 10-year risk, n (%)**			
	Very low risk (<1%)		7 (0.2)
	Lower risk (<median risk or 1-7%)		1714 (47)
	Higher risk (≥median risk or 8-30%)		1630 (45)
	Very high risk (>30%)		279 (8)
**Risk estimate factors** ^a^			
	**HDL (“good”) cholesterol (mg/dL), n (%)**		
		<35	160 (4)
		35-44	304 (8)
		45-49	218 (6)
		50-59	231 (6)
		≥60	321 (9)
		I don’t know	2396 (66)
	**Total cholesterol (mg/dL), n (%)**		
		<160	566 (16)
		160-199	622 (17)
		200-239	368 (10)
		240-279	70 (2)
		≥280	24 (1)
		I don’t know	1980 (55)
	**Systolic blood pressure (mm Hg), n (%)**		
		<120	989 (27)
		120-129	1095 (30)
		130-139	478 (13)
		140-149	186 (5)
		150-159	59 (2)
		≥160	36 (1)
		I don’t know	789 (22)
	Currently taking medication to treat high blood pressure, n (%)		1182 (33)
	Has diabetes, n (%)		469 (13)
	Has smoked in the past month, n (%)		974 (27)
	**Body mass index (BMI), n (%)**		
		<18 (underweight)	30 (1)
		18-24.9 (normal weight)	690 (19)
		25-29.9 (overweight)	794 (22)
		≥30 (obese)	932 (26)
		Height and/or weight not given^b^	1184 (33)
**Other individual difference measures**			
	Subjective numeracy (out of possible 6-48), median (IQR)		35 (10)
	Knows someone who died because of heart problems, n (%)		2702 (75)

^a^HDL: high-density lipoprotein.

^b^Height and weight were only asked of participants who did not know their cholesterol counts.

### Primary Outcome: Risk Perception

We first explored relationships between actual risk and risk perception via Pearson correlations, stratifying by all possible combinations of design factors as shown in Table 2. We observed that correlation values appear to be larger overall in the random condition than in the standard condition, and that nonmoving avatars also appeared to possibly increase correlations.

Testing the risk graphic factors and moderators for their effects on risk perception, we observed an interaction between the actual risk and the random variables in their association with risk perception. Adding the element of randomness resulted in lower risk feeling smaller, higher risk feeling slightly larger, and very high risk feeling larger (see details in Table 3).

**Table 2:** Correlations between actual risk and risk perception by study arm.

Type of Avatar	Standard		Random	
	*r*	*P*	*r*	*P*
No avatar	.13	.02	.25	<.001
Avatar moves: no; color choice: no	.25	<.001	.30	<.001
Avatar moves: no; color choice: yes	.23	<.001	.18	<.001
Avatar moves: yes; color choice: no	.13	.01	.28	<.001
Avatar moves: yes; color choice: yes	.11	.03	.21	<.001

**Table 3:** Summary of findings for primary outcome risk perception.

Effects				Mean values^a^ (SD)	*F*_1,3576_	*P*
**Effects of experimental design factors**						
	**Interaction between actual risk and random**				6.12	.01^b^
		**Lower risk**				
			Standard	3.2 (2.1)		
			Random	3.0 (2.2)		
		**Higher risk**				
			Standard	3.7 (2.0)		
			Random	3.8 (2.1)		
		**Very high risk**				
			Standard	4.1 (2.1)		
			Random	4.6 (1.9)		
	**Main effect of avatar**				4.61	0.03^b^
		No avatar		3.3 (2.1)		
		Avatar		3.5 (2.2)		
	**Interaction between avatar and familiarity**				5.88	.02
		**No familiarity**				
			No avatar	2.7 (2.0)		
			Avatar	3.2 (2.1)		
		**Familiarity**				
			No avatar	3.5 (2.1)		
			Avatar	3.6 (2.1)		
	**Interaction between avatar, color choice, and blood pressure known**				4.57	.03
		**Blood pressure unknown**				
			No avatar	3.5 (2.2)		
			Generic avatar	3.3 (2.2)		
			Avatar with color choice	3.6 (2.2)		
		**Blood pressure known**				
			No avatar	3.3 (2.1)		
			Generic avatar	3.6 (2.1)		
			Avatar with color choice	3.5 (2.1)		
**Effects of moderating variables**						
	**Main effect of actual risk**				166	<.001
		Lower risk		3.1 (2.2)		
		Higher risk		3.7 (2.1)		
		Very high risk		4.4 (2.0)		
	**Main effect of numeracy**				86.2	<.001
		Low numeracy		3.8 (2.1)		
		High numeracy		3.2 (2.1)		
	**Main effect of familiarity**				28.3	<.001
		No familiarity		3.1 (2.1)		
		Familiarity		3.6 (2.1)		
	**Interaction between actual risk and blood pressure known**				7.56	.006
		**Lower risk**				
			Blood pressure unknown	3.3 (2.2)		
			Blood pressure known	3.0 (2.1)		
		**Higher risk**				
			Blood pressure unknown	3.5 (2.1)		
			Blood pressure known	3.8 (2.0)		
		**Very high risk**				
			Blood pressure unknown	4.3 (2.0)		
			Blood pressure known	4.4 (2.0)		
	**Interaction between numeracy and blood pressure known**				4.36	.04
		**Lower numeracy**				
			Blood pressure unknown	3.6 (2.1)		
			Blood pressure known	3.8 (2.1)		
		**Higher numeracy**				
			Blood pressure unknown	3.2 (2.3)		
			Blood pressure known	3.2 (2.1)		

^a^Assessed on scale of 0 (lowest risk perception) to 9 (highest risk perception).

^b^No longer significant when participants at very low or very high risk were removed from the sample.

We observed a main effect for the design factor avatar, with slightly higher overall risk perceptions when an avatar was used, as well as another interaction between avatar and familiarity on this outcome. This interaction suggested that for people who knew someone who had died of cardiovascular problems, the use of an avatar was associated with a minimal increase in risk perceptions, but for people who lacked such familiarity, an avatar significantly increased their risk perceptions.

As expected, all 3 planned moderators had significant main effects, with risk perception increasing with actual risk and decreasing with increasing numeracy. People who knew someone who had died of cardiovascular problems (familiarity) perceived their risk as larger. Neither additional moderator (blood pressure known and cholesterol known) had a significant main effect on risk perception. There was, however, a significant interaction between blood pressure known and actual risk on this outcome. Among participants at lower risk, knowing one’s blood pressure was associated with lower risk perception whereas the reverse was true for participants at higher risk and, to a certain extent, those at very high risk. We also observed an interaction between blood pressure known and numeracy. For participants with higher numeracy, knowing one’s blood pressure did not appear to affect risk perception whereas for those with lower numeracy, knowing one’s blood pressure was associated with somewhat higher risk perception.

Finally, we observed an interaction between blood pressure known, avatar, and color choice in their association with this outcome. Among people who knew their blood pressure, the presence of a generic avatar was associated with somewhat higher risk perception but no additional increase was observed for a personalized avatar. However, among people who did not know their blood pressure, a generic avatar was associated with a small decrease in risk perception whereas a personalized avatar was associated with a small increase.

When we explored these analyses on the middle 2 subsets of participants, removing all participants with risk estimates less than 1% or greater than 30%. Findings remained similar overall; however, the observed interaction between actual risk and random was no longer significant (*F*_1,3291_=2.58, *P*=.10) nor was the observed main effect of avatar (*F*_1,3291_=3.62, *P*=.06).

### Secondary Outcomes

#### Lifestyle Intentions

Examining the effects of different variables on lifestyle intentions, we observed that the factor random had a main effect: participants who received randomly dispersed events were less likely to indicate intentions toward healthy behaviors in the next 30 days (see details in Table 4).

In addition, nearly all moderating variables had significant main effects. Greater intentions toward healthy lifestyles were observed among participants with lower actual risk, those with higher numeracy, and those who knew their blood pressure and cholesterol.

No significant interactions were observed on this outcome. When we explored these analyses within the subgroup of participants that remained after removing all participants with risk estimates less than 1% or greater than 30%, all findings remained similar.

**Table 4:** Summary of findings for secondary outcome lifestyle intentions.

Effects			Mean values^a^ (SD)	*F*_1,3572_	*P*
**Effects of experimental design factors**					
	**Main effect of random**			11.1	<.001
		Standard	5.2 (2.2)		
		Random	4.9 (2.2)		
**Effects of moderating variables**					
	**Main effect of actual risk**			17.4	<.001
		Lower risk	5.2 (2.2)		
		Higher risk	5.0 (2.3)		
		Very high risk	4.7 (2.1)		
	**Main effect of numeracy**			25.4	<.001
		Lower numeracy	4.9 (2.2)		
		Higher numeracy	5.2 (2.2)		
	**Main effect of blood pressure known**			30.8	<.001
		Blood pressure unknown	4.7 (2.4)		
		Blood pressure known	5.2 (2.2)		
	**Main effect of cholesterol known**			34.9	<.001
		Cholesterol unknown	4.8 (2.3)		
		Cholesterol known	5.4 (2.1)		

^a^Assessed on scale of 0 (lowest intentions) to 9 (highest intentions).

#### Intentions to See a Doctor

We observed a significant interaction between actual risk and avatar in which the use of an avatar appeared to increase the spread, making those at lower risk less likely to plan to see a doctor, and those at higher risk more likely (see details in Table 5).

The 3 moderating variables having to do with medical data all had significant main effects in expected directions on participants’ intentions to see a doctor in the next 30 days. Participants with higher actual risk indicated stronger intentions, as did those who knew their blood pressure and cholesterol.

We observed another interaction between numeracy, avatar, and color choice. Among those with higher numeracy, personalization via color choice was associated with somewhat increased intentions to see a doctor, whereas this difference was not observed for those with lower numeracy.

When we explored these analyses within the subgroup of participants that remained after removing all participants with risk estimates <1% or >30%, all findings described previously remained similar; however, an interaction that did not reach significance in the analysis of the full dataset (*P*=.06) was significant in the restricted dataset. Specifically, within the restricted set, we observed an interaction between actual risk and blood pressure known (*F*_1,3273_=6.73, *P*=.01). Among those who did not know their blood pressure, intentions to see a doctor were similar across levels of risk, whereas for those who knew their blood pressure, increased risk was associated with increased intentions (blood pressure unknown: mean 4.2 (SD 3.0) for lower risk vs mean 4.1 (SD 3.2) for higher risk; blood pressure known: mean 4.5 for lower risk (SD 3.0) vs mean 5.4 (SD 2.9) for higher risk).

**Table 5:** Summary of findings for secondary outcome see a doctor.

Effects				Mean values^a^ (SD)	*F*_1,3556_	*P*
**Effects of experimental design factors**						
	**Interaction between avatar and actual risk**				6.38	.01
		**Lower risk**				
			No avatar	4.7 (3.0)		
			Avatar	4.4 (3.0)		
		**Higher risk**				
			No avatar	4.8 (2.9)		
			Avatar	5.2 (3.0)		
		**Very high risk**				
			No avatar	4.9 (3.4)		
			Avatar	5.8 (2.6)		
	**Interaction between numeracy, avatar, and color choice**				10.1	.001
		**Lower numeracy**				
			No avatar	4.8 (2.9)		
			Generic avatar	4.9 (3.0)		
			Avatar with color choice	4.8 (3.0)		
		**Higher numeracy**				
			No avatar	4.7 (3.0)		
			Generic avatar	4.8 (3.0)		
			Avatar with color choice	5.1 (3.1)		
**Effects of moderating variables**						
	**Main effect of actual risk**				81.6	<.001
		Lower risk		4.5 (3.0)		
		Higher risk		5.1 (3.0)		
		Very high risk		5.7 (2.8)		
	**Main effect of blood pressure known**				44.5	<.001
		Blood pressure unknown		4.2 (3.1)		
		Blood pressure known		5.0 (3.0)		
	**Main effect of cholesterol known**				63.0	<.001
		Cholesterol unknown		4.4 (3.1)		
		Cholesterol known		5.4 (2.9)		

^a^Assessed on scale of 0 (lowest intentions) to 9 (highest intentions).

#### Recall

We observed an interaction between random and actual risk in their association with recall. Participants at lower risk demonstrated a slight increase in correct recall in the random condition, those at higher risk showed a slight decrease, and those at very high risk had a larger decrease (see details in Table 6).

Differences in numeracy were also associated with differences in recall. Participants with lower numeracy had more trouble accurately recalling their risk estimate. We also observed a similar main effect for blood pressure known. Participants who knew their blood pressure were also more able to recall their risk estimate.

Rerunning these analyses after removing the participants who had received a less precise estimate in the text (ie, those who received an estimate of “less than 1%” or “more than 30%” but who nonetheless received a risk graphic with a discrete number of event rectangles), we found that the main effects of moderating variables remained similar, but the interaction between random and actual risk was no longer significant (*F*_1,3260_=4.73, *P*=.03). There was also a complex interaction between random, avatar, and avatar moves that did not reach significance in the larger dataset (*F*_1,3544_=5.16, *P*=.02) but did so in the restricted set (*F*_1,3260_=6.27, *P*=.01). In this interaction, we observed that in the standard condition, presence of an avatar was associated with a decrease in correct recall, more so with a moving avatar (correct recall: 85% without avatar, 82% with nonmoving avatar, 78% with moving avatar). On the other hand, in the random condition, correct recall was somewhat lower to begin with and remained relatively consistent, regardless of the presence or absence of an avatar and whether or not it moved randomly within the graphic (correct recall: 80% without avatar, 80% with nonmoving avatar, 79% with moving avatar). Table 7 presents participants’ recall by study arm.

**Table 6:** Summary of findings for secondary outcome recall.

Effects				Participants with correct recall^a^	*F*_1,3544_	*P*
**Effects of experimental design factors**						
	**Interaction between random and actual risk**				7.06	.008^b^
		**Lower risk**				
			Standard	83%		
			Random	85%		
		**Higher risk**				
			Standard	79%		
			Random	76%		
		**Very high risk**				
			Standard	76%		
			Random	64%		
**Effects of moderating variables**						
	**Main effect of numeracy**				75.7	<.001
		Lower numeracy		74%		
		Higher numeracy		86%		
	**Main effect of blood pressure known**				10.6	.001
		Blood pressure unknown		74%		
		Blood pressure known		82%		

^a^Analysis used quasi-continuous difference between recalled value and actual risk. Correct recall for reporting purposes defined as within 5 percentage points.

^b^No longer significant when participants at very low or very high risk were removed from the sample.

**Table 7:** Percent correct recall^a^ by study arm.

Type of Avatar	All data included (n=3597)		<1 and >30 removed (n=3312)	
	Standard	Random	Standard	Random
No avatar	85%	80%	85%	82%
Avatar moves: no, color choice: no	86%	82%	86%	82%
Avatar moves: no, color choice: yes	78%	77%	78%	78%
Avatar moves: yes, color choice: no	78%	83%	79%	84%
Avatar moves: yes, color choice: yes	77%	75%	78%	78%

^a^Correct recall for reporting purposes defined as recall within 5 percentage points of given estimate.

## Discussion

### Principal Results

Our results demonstrate several key findings. First, consistent with our earlier work [29], by animating event rectangles in an icon array to appear one at a time scattered randomly throughout the array and then having them settle at the bottom of the array, we were able to convey the randomness of events without sacrificing overall indications of comprehension of the risk estimate. Further, this design factor helped to increase the concordance between actual risk and risk perceptions, with people at lower risk perceiving themselves to be at lower risk, and people at higher risk perceiving themselves at higher risk. This suggests that animated randomness of this sort may help people better understand their individual risk. It may be that random dispersion of the colored rectangles may reinforce the rareness of small risks and also the magnitude of large ones (eg, “There are colored blocks everywhere!”) This finding is especially notable given that we did not provide participants with any indication of whether their risk was low or high.

The observed effect of animated randomness was driven by the strength of the effect for the 8% of participants who were at very high risk and whose risk estimate was presented in text as “more than 30%.” These participants had to wait as 31 event rectangles appeared randomly in the graphic one by one, never quite knowing where the next one would appear or when the process would stop. The combined uncertainty of the text statement, random positioning of the event rectangles, and uncertainty around how many event rectangles would appear may well compound each other and lead to a heightened sense of being at risk. Because it is common for models of health risk to be mathematically convergent only within certain boundary conditions and/or to generate ranges of risk estimates rather than point estimates, this design technique of animated randomness combined with the temporal signaling of one event appearing at a time may be broadly applicable. Nonetheless, further research will be needed to determine its effectiveness—or lack thereof—across a range of situations.

We further note that although there was no main effect of animated randomness on recall, those at higher risk demonstrated a small decrease in their ability to precisely recall the risk estimate they were given whereas those at lower risk demonstrated a small increase. As with the interaction discussed previously, this relationship was driven by the strength of effect in those at very high risk, who were not given a precise numerical risk estimate in text and thus had the more challenging task of recalling the number of event rectangles in their risk graphic. This additional difficulty may have contributed to the lower recall in this group. We further speculate that people who are reassured by a risk estimate may find it somewhat easier to remember the number whereas those who are alarmed may be less likely to remember an exact number because of distracting emotions, such as fear.

Despite the overall welcome finding that animated randomness may help better align risk perceptions with actual risk, we note that in the study context of lifestyle-preventable disease, emphasizing the haphazard and random distribution of negative outcomes led to lower intentions of behavior that might prevent such events. Visually depicting randomness may cause people to focus on the role of chance in health outcomes, drawing their attention to the fact that one’s behavior does not completely determine one’s health outcomes. We note that we did not present visual depictions of the potential for health behaviors to change the risk estimate. Doing so might possibly have reduced the negative effects of animated randomness on healthy behavior intentions. Further research will be necessary to fully understand this aspect of our findings. Such research should explore the role of potential moderators of health behavior, such as beliefs about the efficacy of lifestyle changes as well as self-efficacy and fatalism in health.

Ultimately, these findings suggest that the value of explicitly showing randomness may depend on whether one’s goal is to persuade or inform. Helping people understand randomness may be less useful in persuasive contexts such as promoting lifestyle change. However, it may be more useful in cases in which the primary objective is to inform; for example, in preference-sensitive decisions or when informing people about the risk of a side effect. Importantly, aiming to fully inform people is arguably more ethical than aiming to persuade them. This design factor will need to be tested in other contexts and may also require some unpacking to determine the effects of design choices that did not vary across experimental conditions, such as the fact that higher risks took a longer time to appear.

Second, using an avatar increased overall risk perceptions, and showed promise particularly for people who did not personally know anyone who had experienced grave outcomes in this context. Thus, avatars may be especially useful for drawing attention to risks related to rare or hidden conditions. This interaction, combined with the fact that the main effects of familiarity were similar to the effects of an avatar in the absence of familiarity, suggests that the avatar achieved our design goal of helping people better grasp how population-based statistics can apply to an individual. This conclusion is bolstered by the fact that use of an avatar significantly shifted intentions to see a physician in sensible directions, with people at lower and higher risk indicating, respectively, lower and higher intentions to see a doctor in the next 30 days.

However, other design factors related to the presentation of the avatar showed mixed results. Having the avatar move around randomly in the risk graphic appeared to simply confuse most participants. Allowing people to choose the color of their avatar may have increased identification with the avatar among those with higher numeracy, as they were more likely to indicate intentions to see a doctor when they were encouraged to choose the color. We speculate that those with higher numeracy may be better equipped to understand the risk graphic and thus, adding an extra element that draws their attention can be helpful. By contrast, the extra factor may have added a level of confusion or overwhelm for those with lower numeracy. Allowing people to choose the color of their avatar also appeared to make up for the loss of personal salience among participants who did not know their blood pressure and thus, slightly encouraged intentions to engage in health lifestyle behaviors and see a doctor. However, for those who knew their blood pressure, it tended to have the opposite effect. Taken together, these results suggest that when risk salience may be low, using a personalized avatar may help people feel like the risk applies to them, individually. However, these effects were small; moreover, if risk salience is higher, such basic attempts at personalization may backfire. Therefore, using color choice as a method of personalization, although efficient and quick, appears to be insufficient to allow all people to identify with their avatar and may even lead to undesirable results. Further research will be required to investigate the effects of different forms of personalization.

Third, we note that all 3 planned moderating factors had significant effects. For example, people at higher risk perceived their risk as higher and indicated stronger intentions to see a doctor in the next 30 days; people scoring lower on numeracy indicated higher overall risk perceptions, lower intentions toward healthy behaviors, and lower recall, and participants who knew someone who had died of cardiovascular causes had higher risk perceptions. We also observed that people at higher risk tended to indicate lower intentions toward healthy behavior. This latter finding—that people at higher risk have lower intentions to do such things as quit smoking, exercise, and eat well in the next 30 days—may reflect that those at lower risk may already be engaging in those behaviors and thus can easily indicate higher intentions toward such behaviors in the next 30 days. It also aligns with findings about how negative feedback can discourage people, whereas positive feedback can motivate people in a success breeds success cycle [52]. These findings support the need for extra attention when considering how to design better risk communication for those at higher risk, lower numeracy, and with more or less personal familiarity with the condition. We highlight that the fact that many of the observed interactions with actual risk were driven by the participants at the highest level of risk suggests that risk communication methods should be tested across the full range of possible risk values to ensure that studies capture relevant findings.

Analyses of participants who did or did not know their blood pressure and/or cholesterol suggested that these were important moderators, particularly the former. People who knew their blood pressure had greater alignment between their actual and perceived risk, overall higher intentions toward healthy lifestyle actions and seeing a doctor, and more accurately recalled their risk estimate. It may be that this latter finding is reflective of an underlying ability to recall numbers; however, in such a case, we would expect to see an interaction with numeracy, which was not present. Numeracy did interact with knowledge of one’s blood pressure when it came to risk perceptions and behavioral intentions. For people with higher numeracy, risk perception was consistent whether they knew their blood pressure or not, whereas for those with lower numeracy, their overall higher risk perceptions were further increased with knowledge of their blood pressure. In addition, for those with lower numeracy, knowing one’s blood pressure was more influential in increasing behavioral intentions than it was for those with higher numeracy. We speculate that people with lower numeracy may accord more importance to their blood pressure number. Similar to the results for blood pressure, people who knew their cholesterol were more likely to indicate intentions to engage in healthy behaviors and see a doctor in the next 30 days. These findings support the idea that risk estimates are likely to be more impactful when they are more individually tailored.

### Limitations

This study was limited by the fact that we used an Internet survey panel to recruit participants. Although this recruitment choice allows us to ensure a more diverse sample, it necessarily introduces selection bias in that participants are those who registered on a panel to take surveys; thus, they may not be representative of the broader population.

In addition, we observed small effects. This is expected in a study with outcomes such as risk perceptions and behavioral intentions because such outcomes have significant variation in individual responses. Even the actual risk estimate was associated with only a 1.4-point difference on a 10-point scale of risk perception between those at high and very low risk, suggesting that there is little room on the scale within which to work. This limits the overall utility of these design factors, as it may not be worth the additional design complexity and development time to make small gains. Such small gains, however, may be worth pursuing when one considers cumulative effects within a population.

The underlying risk model also limited this study in 2 ways. First, because the risk estimate for cardiovascular disease uses age as an important predictor, we were not able to isolate the effects of age on participants’ reactions to the different risk communication designs. It is possible, for example, that older adults might have different reactions to randomization or avatars. Further research will be needed to explore the effects of these kinds of design factors in older versus younger adults. Second, because the model does not provide numerical risk estimates below 1% or above 30%, approximately 8% of the sample population in this study did not receive a precise numerical text estimate. Instead, these participants received a risk estimate of “less than 1%” (7 participants) or “more than 30%” (279 participants) along with 1 or 31 event rectangles in their risk graphic, respectively. This additional ambiguity in the textual risk estimate appeared to amplify findings regarding randomization in the risk graphic. As discussed previously, this is a realistic portrait of many risk calculators, as many risk analysis models are mathematically convergent only within certain boundary conditions. However, the observed interaction between risk level and animated randomness may not translate to calculators that yield precise estimates across a full range of potential risk.

Finally, it is important to note that because this study was conducted in the United States before the introduction of the Affordable Care Act (“ObamaCare”), findings about participants who did not know their blood pressure or cholesterol and findings concerning intentions to see a doctor may reflect an underlying issue of lack of access to medical care rather than effects that would translate to other settings.

### Comparison With Prior Work

Previous work has suggested that randomly displaying events in an icon array can increase understanding of the random nature of such events, but at the expense of comprehension of the numerical risk estimate [22-27]. Our study, along with our previous work [29], demonstrated that animating the randomly distributed events to ultimately settle into a standard display can convey randomness without such a sacrifice.

A previous study by Ancker and colleagues [31] tested a somewhat similar interface in which participants were shown 1 of 4 risk graphics to display a risk of heart disease and also a risk of infectious disease: a static standard icon array, a static random icon array, an interactive display in which participants switched back and forth between the static standard and static random array, and an interactive display in which participants began with all icons covered and clicked to uncover a static random display. The third of these, which the authors dubbed “switch,” had the most similarities with our random condition in that it alternated between random and standard displays of risk. The key differences between our design of randomness and the switch design were in our use of animation to signal quantity by adding one event rectangle at a time and having the rectangles dynamically settle into a standard display at the conclusion of the animation automatically, unlike the interactive nature of Ancker and colleagues’ design [31].

Another study conducted by Han and colleagues [28] examined different methods for representing randomness by experimentally varying the presentation of a hypothetical risk of colon cancer. After introducing participants to the National Cancer Institute Colorectal Cancer Risk Assessment Tool, they tested 5 representation formats for risk; namely, a static standard icon array, a static random icon array, a dynamic random icon array in which the event rectangles changed location randomly (and did not settle into a standard display at the conclusion of the animation), and text with and without cues about randomness.

In comparing our study to previous work, we note that the studies by both Ancker et al [31] and Han et al [28] used hypothetical contexts and assigned risks that did not vary by participant, a common practice in the field of risk communication research when the goal is to evaluate the utility of a given communication format. In contrast, our study had participants enter their personal information into a risk calculator and receive their actual risk estimate. Both previous studies found no differences in risk perceptions by graphic format. We observed a similar lack of main effect of randomness; however, because our study used varying levels of risk, we were able to observe an interaction in which randomness was associated with lower risk perception at lower levels of risk, and higher risk perception at higher levels of risk, suggesting that randomness helps align risk perceptions with actual risk. We note that both of these previous studies included a measure or method of assessing perceived uncertainty in the risk estimate. We did not measure such an outcome; therefore, we are unable to compare our findings to theirs on that construct.

To the best of our knowledge, ours is the first study to test the effects of including an avatar as a design factor in risk communication graphics. Previous work in other contexts has suggested that people identify strongly with their avatar [34-41]. Our results, which suggest that including an avatar helped increase realization of personal risk, are in line with these findings. More research is needed to study the use of avatars and other embodied agents in health risk communication.

More recent research has also suggested that graphical displays may not always outperform simple percentages or absolute frequencies in risk communication [53] and that their utility may depend on graphical literacy, with people who score low on graphical literacy performing better with numbers alone [54]. Because the data for our study were collected before publication of the graphical literacy scale, we did not measure graphical literacy and were unable to examine this issue. Graphical literacy will be an important moderating factor to include in future research, and the question of whether or not to use graphics when communicating risk remains open.

The present study continues a program of research by our group in which we previously noted that 2 animated displays side by side were problematic [29]. In this study, we deliberately used one signal at a time and did not observe the same deleterious effects of animation and randomness on performance. Our findings about the generally negative effects of a moving avatar, however, continue to encourage careful design when including animation and motion in risk graphics.

### Conclusions

An animated display of risk that adds events one at a time in a randomly dispersed icon array and where they settle at the bottom of the display at the conclusion of the animation may help align risk perceptions with actual risk estimates without sacrificing number sense. This method shows promise for helping people better understand the random nature of risk. Such understanding may come at a cost of discouraging behavioral intentions, suggesting that the use of this method may depend on whether the goal of the risk communication is to persuade or to inform.

The use of an avatar in a risk graphic also shows promise for helping people to grasp how population-based statistics can apply to an individual, particularly in cases when the person does not know anyone who has experienced the outcome under consideration. An avatar that is animated to move randomly within the graphic does not appear to be helpful. Personalization via color choice shows mixed effects, suggesting that personalization of an avatar may be an interesting avenue for further study, but that this particular method of personalization does not appear to be optimal.

## Multimedia Appendix 1

Video of risk graphic (.mp4 version).

## Multimedia Appendix 2

Video of risk graphic (.avi version).

## Multimedia Appendix 3

Secondary study: effects of heart age message.

## Abbreviations

AA: Associate of Arts
AS: Associate of Science
BA: Bachelor of Arts
BS: Bachelor of Science
GED: General Educational Development
HDL: high-density lipoprotein
MA: Master of Arts
MD: Doctor of Medicine
MPH: Master of Public Health
PhD: Doctor of Philosophy
SD: sample standard deviation
SSI: Survey Sampling International
