# Supplementary material for: Animated Randomness, Avatars, Movement, and Personalization in Risk Graphics
Source: J Med Internet Res. 2014 Mar 18;16(3):e80. doi: 10.2196/jmir.2895 (PMC3978557; doi:10.2196/jmir.2895)
Supplement: Supplementary file 3 [file jmir_v16i3e80_app3.pdf]

Multimedia Appendix 3: Effects of Heart Age Message

Introduction

For most authors, our interest with this study was about risk communication formats in general. We used cardiovascular risk because of the availability of a robust model that is widely applicable and gives a wide range of risk estimates. We are interested in developing methods for communicating different risks, most of which are only available as proportions, and therefore tested different graphics for this purpose.

However, because the literature in cardiovascular risk communication has emphatically endorsed the use of the "heart age" message, going so far as to recommend it in national guidelines as a means for health care providers to effectively communicate with patients about their cardiovascular health [55], we included a secondary research question to that effect. Previous research has suggested that the heart age message may be more effective than a percentage risk for motivating behavior change [56]. Graphics such as simple icon arrays and bar graphs have been noted as, "confusing and uninspiring," whereas a display of cardiovascular age is, "a wake-up call." [57] The heart age message has been used within a multifactorial and highly effective clinic-based program [58,59] and similarly effective results have been observed in the context of smoking cessation by telling people their "lung age." [60]

Thus, within our larger study, we also conducted a small secondary study in which we cross-randomized all participants to either receive or not receive a message about their "heart age" after completing our primary outcome but before answering secondary outcome measures about behavioral intentions. This was not a primary question in this study; therefore, we describe and report the results of this secondary study here in Multimedia Appendix 2 for interested readers.

Methods

After participants answered questions about risk perceptions, they were randomly assigned to either receive or not receive their, "heart age." For example, a 51 year old woman might receive the message, "In addition to your risk of heart disease and stroke, the calculator can also calculate your 'heart age.' According to the calculator, right now, you have the heart of an average 45 year old woman." When randomized to this condition, this message appeared at the top of the page asking participants about their intentions to engage in certain behaviors (quitting smoking, exercising, eating a DASH diet, losing weight, and seeing a doctor) in the next 30 days. Participants not randomized to this condition saw the same page of behavioral intention questions but without the brief heart age message at the top.

Analysis

To examine the effects of this additional independent variable, *Heart Age Given*, we re-ran the nested factorial ANOVA on behavioral intention outcomes, adding the main

effect of *Heart Age Given* as well as two-way interactions between Heart Age Given and each moderator.

Results

No significant main effect or interactions were observed for *Heart Age Given* on either *Lifestyle Intentions* or intentions to *See a Doctor*. Including this variable in the model slightly altered F-statistics and p-values but did not change findings regarding significance or nonsignificance of other factors. Tables 2-1 and 2-2 present details.

Table 2-1. Effects of heart age given on lifestyle intentions.

Mean (SD)	Heart age not given: mean values[a] (SD)	Heart age given: mean values[a] (SD)	$F_{1,3556}$	*P*
**Overall (main effect)**	5.1 (2.2)	5.0 (2.2)	0.22	.64
Actual Risk **(interaction)**			2.16	.14
Lower (1-7%)	5.2 (2.2)	5.2 (2.2)		
Higher (8-30%)	5.0 (2.3)	5.0 (2.2)		
Very High (>30%)	4.9 (2.1)	4.5 (2.1)		
Numeracy **(interaction)**			1.40	.24
Low	5.0 (2.2)	4.9 (2.3)		
High	5.2 (2.2)	5.2 (2.2)		
Familiarity **(interaction)**			0.00	.95
No Familiarity	4.9 (2.3)	4.9 (2.3)		
Familiarity	5.1 (2.2)	5.1 (2.2)		
Blood Pressure Known **(interaction)**			0.00	.99
Blood Pressure Unknown	4.7 (2.4)	4.7 (2.3)		
Blood Pressure Known	5.2 (2.1)	5.1 (2.1)		
Cholesterol Known **(interaction)**			0.30	.57
Cholesterol Unknown	4.8 (2.3)	4.7 (2.3)		
Cholesterol Known	5.4 (2.0)	5.4 (2.1)		

[a] Assessed on scale of 0 (lowest intentions) to 9 (highest intentions).

Table 2-2. Effects of heart age given on intentions to see a doctor.

Effects	Heart age not given: mean values[a] (SD)	Heart age given: mean values[a] (SD)	$F_{1,3550}$	*P*
**Overall (main effect)**	4.9 (3.0)	4.8 (3.0)	0.41	.52
***Actual Risk* (interaction)**			0.86	.35
Lower (1-7%)	4.5 (3.0)	4.4 (3.0)		
Higher (8-30%)	5.1 (3.0)	5.1 (3.0)		
Very High (>30%)	5.9 (2.8)	5.4 (2.8)		
***Numeracy* (interaction)**			0.42	.52
Low	4.8 (3.1)	4.8 (2.9)		
High	4.9 (3.0)	4.9 (3.0)		
***Familiarity* (interaction)**			3.41	.06
No Familiarity	4.6 (3.0)	4.9 (3.1)		
Familiarity	5.0 (3.1)	4.8 (3.0)		
***Blood Pressure Known* (interaction)**			0.19	.66
Blood Pressure Unknown	4.2 (3.1)	4.2 (3.0)		
Blood Pressure Known	5.1 (3.0)	5.0 (3.0)		
***Cholesterol Known* (interaction)**			0.22	.64
Cholesterol Unknown	4.4 (3.1)	4.4 (3.0)		
Cholesterol Known	5.4 (2.9)	5.3 (2.9)		

[a] Assessed on scale of 0 (lowest intentions) to 9 (highest intentions).

Discussion

Contrary to previous research, we observed no effects of the inclusion of a heart age message. Because we did not directly evaluate the heart age message versus our risk presentation methods, we cannot discuss the relative utility of one method over the other. We offer, however, some comments related to this issue.

First, aside from one focus group study, previous comparative research has tended to compare heart age to simple percentages, whereas our study used best practice graphics developed through considerable trial and error. Second, we included the heart age message after presenting an attention-getting risk graphic, which likely reduced the available variance that could have been accounted for by whether the heart age message was present or absent. Third, previous research has used different messages that differ from one study to the next, but, in general, they compare the participant to someone of a certain age, “without risk factors.” Because the fact that risk factors are influential but not deterministic can be a confusing concept, for reasons of understandability and to avoid potential confounding that might result from having a section explaining what risk factors are and how they influence one’s chances of cardiovascular disease, we used a modified version of the heart age message (see above for exact wording.) Although our use of “average” rather than “without risk factors” was in line with beliefs that the average person is not someone with cardiovascular disease, it was, nonetheless, less accurate and specific, and thus it is possible that we might have had different results had we included a discussion of risk factors and been more specific with our wording. However, given the strong lack of effect on behavioral intentions that we observed, it seems unlikely that this would have appreciably influenced results. This is especially true given that we provided participants with only their absolute number and no indication of whether their risk was high or low before they saw the heart age message. This should have increased the impact of the heart age message particularly for those who were at higher risk, yet we observed no such effect. Fourth and finally,

the strongest evidence for the heart age message format has been obtained in face-to-face encounters with a clinician, which is a more powerful context than an anonymous online survey.

The heart age frame certainly has significant face validity. One would think that receiving a heart age that is significantly above or below one's actual age would be a compelling message. However, our results suggest that such a message does not add significantly to the existing impact of a risk graphic. To truly answer the question of the effectiveness of best practices in risk graphics versus a given "[organ] age," further research will be required.

Additional References for Multimedia Appendix 2

N.B. For coherence, these also appear in the main references section.

55. Rabi DM, Daskalopoulou SS, Padwal RS, Khan NA, Grover SA, et al. (2011) The 2011 Canadian Hypertension Education Program Recommendations for the Management of Hypertension: Blood Pressure Measurement, Diagnosis, Assessment of Risk, and Therapy. CJCA 27: 415–433.e2. doi:10.1016/j.cjca.2011.03.015.

56. Soureti A, Hurling R, Murray P, van Mechelen W, Cobain M (2010) Evaluation of a cardiovascular disease risk assessment tool for the promotion of healthier lifestyles. European Journal of Cardiovascular Prevention & Rehabilitation 17: 519–523. doi:10.1097/HJR.0b013e328337ccd3.

57. Goldman RE, Parker DR, Eaton CB, Borkan JM, Gramling R, et al. (2006) Patients' perceptions of cholesterol, cardiovascular disease risk, and risk communication strategies. The Annals of Family Medicine 4: 205–212. doi:10.1370/afm.534.

58. Grover SA, Lowensteyn I, Joseph L, Kaouache M, Marchand S, et al. (2007) Patient knowledge of coronary risk profile improves the effectiveness of dyslipidemia therapy: the CHECK-UP study: a randomized controlled trial. Archives of Internal

Medicine 167: 2296–2303. doi:10.1001/archinte.167.21.2296.

59. Grover SA, Lowensteyn I, Joseph L, Kaouache M, Marchand S, et al. (2008) Discussing Coronary Risk with Patients to Improve Blood Pressure Treatment: Secondary Results from the CHECK-UP Study. J Gen Intern Med 24: 33–39. doi:10.1007/s11606-008-0825-4.

60. Parkes G, Greenhalgh T, Griffin M, Dent R (2008) Effect on smoking quit rate of telling patients their lung age: the Step2quit randomised controlled trial. BMJ 336: 598–600. doi:10.1136/bmj.39503.582396.25.
